# Periapically Extruded Calcium Silicate Cements in Retreated Teeth: A Case Report and Literature Review

**DOI:** 10.1155/crid/9045789

**Published:** 2025-03-28

**Authors:** Jelena Vidas Hrstić, Mario Jakobović, Sanja Šegović, Gabriel Tokić, Ivana Brekalo Pršo

**Affiliations:** ^1^Department of Endodontics and Restorative Dentistry, Faculty of Dental Medicine, University of Rijeka, Rijeka, Croatia; ^2^Department of Dental Medicine, Faculty of Dental Medicine and Health, Josip Juraj Strossmayer University of Osijek, Osijek, Croatia; ^3^Department of Endodontics and Restorative Dentistry, School of Dental Medicine, University of Zagreb, Zagreb, Croatia; ^4^Faculty of Dental Medicine, University of Rijeka, Rijeka, Croatia

**Keywords:** bioceramic sealer, calcium silicate cements, mineral trioxide aggregate, root canal retreatment, sealer extrusion

## Abstract

Calcium silicate–based materials, known for their bioactive properties, are increasingly being used in endodontic therapy. Accidental extrusion of these materials into the periapical tissue is a relatively common phenomenon that can impede periapical healing. The aim of this article is to present three endodontically retreated case reports with moderate to extensive periapical lesions and to review the literature on this topic. The comprehensive search and analysis of the relevant literature included a review of 51 articles, of which nine case reports/series from 2010 to 2023 were considered. Based on the summarized data, over 80% of the retreated cases showed complete healing of the periapical lesions, while the success rate in initially treated teeth was over 90%. Healing of periapical lesions is the most common therapeutic outcome, even in cases where extrusion occurs. Despite this conclusion, extrusion of materials into periapical tissues should be avoided. Further case reports on the extrusion of calcium silicate–based materials other than MTA are recommended. When evaluating the success rate of periapical healing in such cases, it is recommended to extend the follow-up periods to several years.

## 1. Introduction

Endodontic therapy is a method of choice for teeth with pulpal/periapical disease to prevent extraction. However, according to various authors, the cumulative success rate of endodontic treatment is between 25% and 85% [1–3].

This wide variation in success rate may be attributed to several factors, including incomplete eradication of microorganisms and their by-products, the difficulty of locating all root canals (RCs) within the complicated endodontic system of a single tooth, inadequate coronal sealing, and, most importantly, inadequate sealing of the apical portion [4–7].

Therefore, the general assumption that the outcome of endodontically retreated teeth is even worse compared to healing compared with initially endodontically treated teeth is not surprising [8, 9]. In cases where clinical symptoms persist and there is a potential lack in healing of periapical lesions, the retreatment of endodontic filling is necessary to prevent periapical surgical intervention or extraction [10].

The placement of apical plugs in the treatment of teeth with open apices may result in the extrusion of cements (and/or gutta-percha) into the periapical tissues, which can lead to a number of complications, including inflammation, pain, and impaired healing with the possibility of permanent tooth loss. Therefore, the selection of suitable obturation material is crucial.

Calcium silicate (CS) cements, also known as bioceramics (BCs), have superior physical and chemical properties compared to conventional sealants due to their biocompatibility, bioactivity, and great sealing capacity in a moist environment [11–13].

CS cements play a role in wound healing and repair the tissue by influencing the behavior of the cells surrounded by this material such as mesenchymal cells, osteoblasts, osteoclasts, and fibroblasts [14]. These materials also have antimicrobial potential due to their high pH and solidify through hydration reactions, forming hydroxyapatite upon prolonged contact with tissue fluids [15].

Thanks to the abovementioned properties, BCs are the material of choice, especially in challenging endodontic cases when it comes to forming apical plugs in teeth with open apices (immature teeth, apicotomized, overinstrumented, etc.).

The beginnings of CS date back to the 1990s, when Torabinejad invented mineral trioxide aggregate (MTA), the gold standard among bioactive materials [16].

Considering the claim by some authors that the healing process of periapical lesions could be impaired by the extrusion of CS, taking into account the observation by Chybowski et al. that periapical lesions smaller than 5 mm in diameter had a significantly higher success rate when treated with CS (EndoSequence) compared to larger lesions, and noting the lack of similar clinical cases, the aim of this article is to provide clinical evidence and a comprehensive literature review on the success rates associated with the healing of large periapical lesions in retreated teeth with inadvertent extrusion of CS-based sealants [17–21].

## 2. Case Report 1

**Figure 1:**
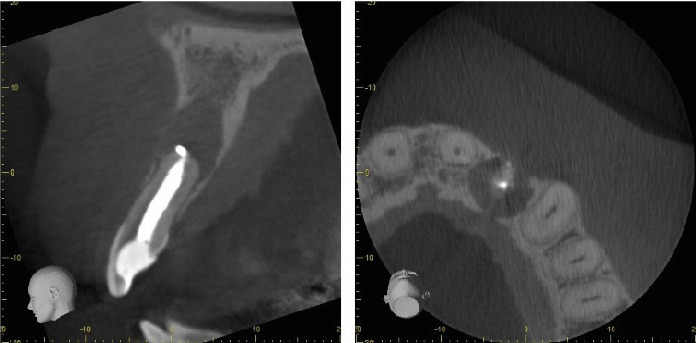
Clinical features of Case Report 1. Preoperative CBCT snapshot of Tooth #22.

**Figure 2:**
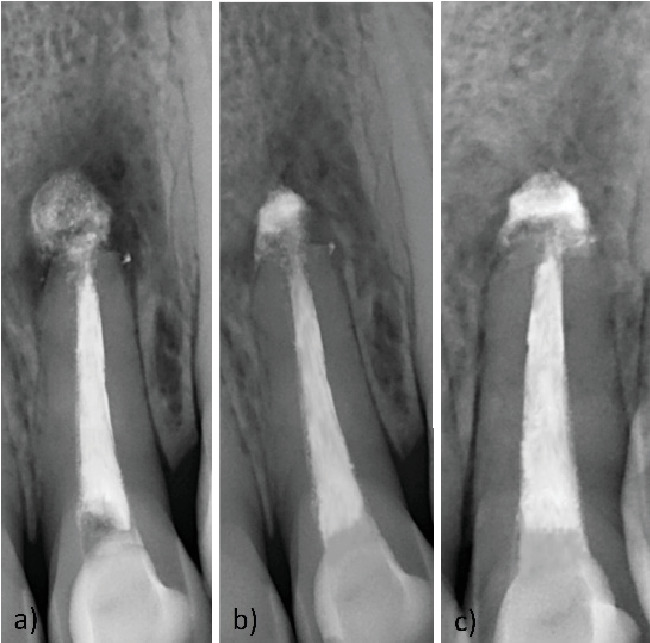
Clinical features of Case Report 1. (a) Postoperative intraoral radiograph, a notable portion of extruded MTA. (b) Six-month follow-up, note gradual healing of the periapical lesion. (c) Twenty-month follow-up, note the periapical osseous healing with gradual resorption and reorganization of extruded MTA.

A female patient (22) was referred to the Department of Endodontics and Restorative Dentistry at the Clinical Hospital Center Rijeka due to the presence of sinus tract in the apical projection of Tooth #22. The patient reported a history of trauma affecting the tooth, which was endodontically treated 10 years ago and apicoectomized 4 years ago. An intraoral examination revealed palpatory hypersensitivity of the vestibular tissue in the apical projection and tenderness on percussion of the same tooth. Discoloration of the crown of the tooth was also visible. The patient was referred to a CBCT, and a chronic apical abscess was diagnosed (Figure 1). Chemomechanical retreatment was performed using the Endo ReStart Ni-Ti rotary file system (Komet, Lemgo, Germany) in combination with stainless steel hand instruments (MAF #60) and passive ultrasonic irrigation (PUI) (EndoUltra System by MicroMega, Coltene, Besancon, France) in combination with 3% NaOCl and 17% EDTA. After the first appointment, a calcium hydroxide (CH) dressing was applied for a 2-week interval, and the sinus tract was completely closed. Apical sealing was performed with MTA (ProRoot MTA, Dentsply Tulsa Dental, Tulsa, Oklahoma), followed by thermoplastic injectable gutta-percha (Elements IC, Kerr, United States) for the rest of the RC at the next appointment (Figure 2). Two follow-up examinations were carried out (although the patient was advised to attend follow-up appointments every 6 months), after 6 (Figure 2) and 20 months (Figure 2). At the follow-up examination after 20 months, the patient was advised to do a follow-up CBCT scan, but due to personal conviction, only a radiological scan was performed. The tooth was asymptomatic and fully functional. After 20 months, the dental crown was internally whitened with 35% hydrogen peroxide (Opalescence Endo, Ultradent, South Jordan, Utah, United States) using the “walking bleach” technique for 4 days.

## 3. Case Report 2

**Figure 3:**
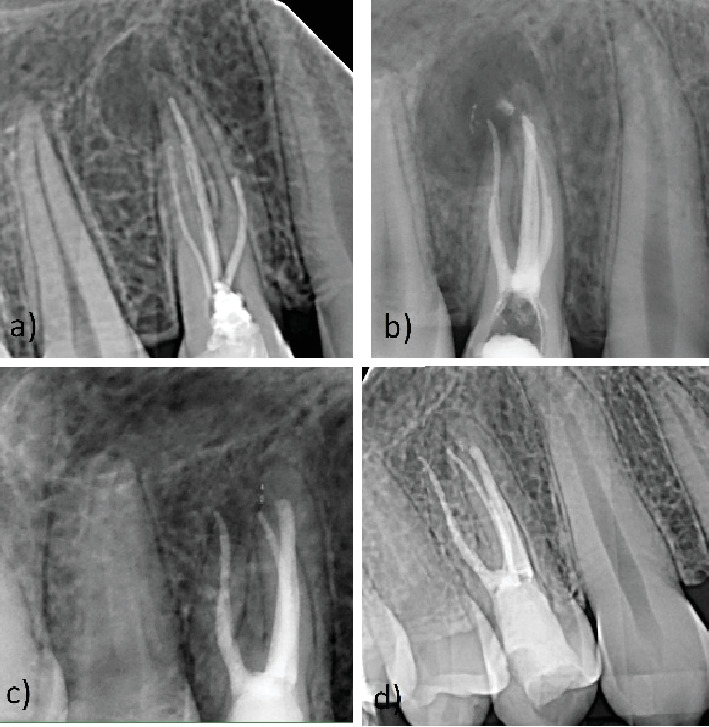
Clinical features of Case Report 2. (a) Preoperative intraoral radiograph. (b) Postoperative intraoral radiograph, a small amount of extruded CS material into the periapical tissue, note the size of the periapical lesion. (c) Twelve-month follow-up, gradual healing of the periapical lesion. (d) Twenty-four–month follow-up, complete healing of the periapical lesion.

A male patient (29) was referred to the same dental teaching hospital with primary concern regarding pain on palpation of the apical region and when biting on Tooth #14. The tooth had been endodontically treated 3 years prior and restored with a composite filling. The intraoral examination confirmed palpable hypersensitivity of the vestibular tissue in the apical region and on vertical percussion. Radiographic assessment revealed molarization with an extensive periradicular lesion involving all three roots and inadequate RC obturation (Figure 3).

Once the operating field was isolated, endodontic retreatment was carried out as single-visit therapy. Chemomechanical treatment was performed with the same system of rotating Ni-Ti and PUI as in the previous case. Obturation was performed with single-cone gutta-percha cones chosen according to the size of the canal preparation (buccal RCs 30/0.4, palatal RC 40/0.4), together with EndoSequence BC Sealer (Brasseler, Savannah, Georgia, United States). The postoperative radiograph showed moderate extrusion of the CS cement (Figure 3b). Subsequent radiographic evaluations 12 and 24 months after treatment showed periapical healing (Figure 3c,d). The tooth remained asymptomatic and fully functional throughout the observation period.

## 4. Case Report 3

**Figure 4:**
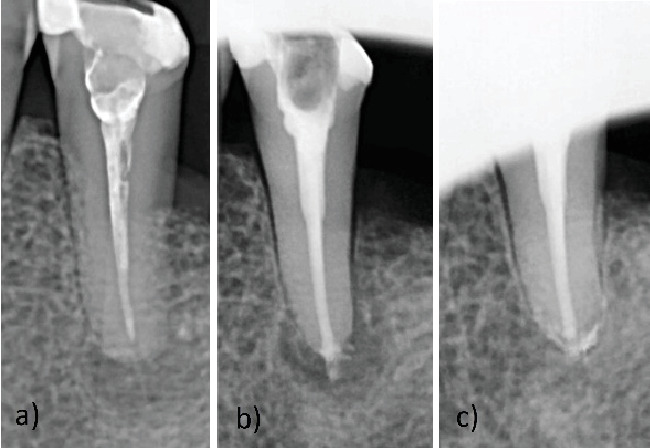
Clinical features of Case Report 3. (a) Preoperative intraoral radiograph. (b) Postoperative intraoral radiograph, moderate extrusion of CS cement. (c) Nineteen-month follow-up, healed periapical lesion with reorganization of the extruded material.

A 70-year-old male patient was referred to the same clinic complaining of discomfort upon biting on Tooth #35, which had been endodontically treated 11 years ago and restored with a large composite filling. The patient's medical history was nonsignificant. On examination, percussion sensitivity and palpable hypersensitivity of the vestibular tissue in the apical region were noted. The preoperative intraoral periapical radiograph showed apical periodontitis with a well-defined radiolucent area (Figure 4a).

After rubber dam isolation, endodontic retreatment was carried out as single-visit therapy. Chemomechanical treatment was performed with the same NI-Ti rotary instruments and PUI system as in previous cases. Obturation was performed with a single gutta-percha cone chosen according to the size of the canal preparation (30/0.4) and EndoSequence BC Sealer. The postoperative radiograph showed a homogeneously filled RC with moderate extrusion of CS cement (Figure 4b). The patient attended only one follow-up appointment 19 months after retreatment. The radiograph showed a healed periapical lesion with reorganization of the extruded material and the patient was completely asymptomatic (Figure 4c). The tooth remained asymptomatic and fully functional during the entire observation period. The patient will continue to come for follow-up examinations.

All patients filled out a consent form from the Rijeka Hospital Clinical Center at the initial examination and agreed to the proposed therapeutic procedures.

## 5. Literature Review

The comprehensive search and analysis of the relevant literature included a review of 51 articles of which 9 were case reports. The research was carried out by searching Medline and PubMed databases, supplemented by individual searches on related topics. In addition, PubMed cross-references were checked to identify further relevant literature. The search included scientific literature retrieved by searching for general terms such as “bioceramic apical extrusion,” “endodontic re-treatment extrusion,” and “apical MTA extrusion” and their various combinations. Only articles written in English were considered for this study. Cases that did not document periapical extrusion of BC sealer, cases with overextended gutta-percha, deciduous teeth, and those that did not specify the type of material extruded or did not provide an exact number of initially treated and retreated cases were excluded from the analysis. In addition, case reports documenting the extrusion of cement into the mandibular canal, resulting in either permanent or transient clinical symptoms, were intentionally excluded from this literature review [22, 23].

## 6. Discussion

In persistent periapical lesions, a conservative approach to endodontic treatment is always advantageous over periapical surgery. In these case reports, conservative retreatment with CS cements was performed, taking into account the size of the periapical lesions and the clinical signs.

Studies in animal models show that CS induces a dense infiltration of inflammatory cells that can be observed within 7 days after subcutaneous implantation. However, this inflammation gradually decreases and completely resolves by the 90th day after implantation [24–26].

Precipitation or biomineralization is triggered by the expression of osteopontin, which is essential for the initial formation of the bone matrix and its calcification osteocalcin and alkaline phosphatase [27–29].

The release of calcium ions from bioactive cements is considered to be the main inductor of osteoblastic differentiation and bone formation [30, 31]. These ions act as extracellular and intracellular messengers that control specific processes such as mitosis, cell death, and cell differentiation. An increased extracellular calcium ion concentration induces chemotaxis and the differentiation of pluripotent cells into osteoblasts via calcium-sensitive receptors [32]. Binding of calcium ions to calcium-sensitive receptors leads to the release of calcium from the endoplasmic reticulum, further increasing intracellular calcium ion concentration, which in turn contributes to osteoblast differentiation [30, 32, 33].

Described findings presented from animal studies are intended to simulate optimal conditions for bone healing. Nevertheless, manipulation of CS in in vivo terms, especially MTA, may be challenging when trying to achieve an apical plug, potentially leading to inadvertent extrusion of material into periapical tissues [34]. Indeed, extrusion of obturating material may impede the healing process of periapical lesions, thereby causing clinical symptoms [35–37].

Specifically, the incorporation of various radiopacifiers, which are necessary to enhance the radiographic visibility of the material, may inadvertently interfere with its setting reactions and bioactive properties, as noted by Camilleri [38]. Furthermore, certain radiopacifiers have been associated with cytotoxic effects when in direct contact with human tissues [39]. Therefore, achieving an optimal balance in radiopacifier content is essential to ensure adequate radiopacity while minimizing any adverse impact on periapical tissues [40].

The most frequently used CS material in this literature review was ProRoot MTA (Dentsply Tulsa Dental, Tennessee, United States) available in both white (tooth-colored) and gray variants, though some authors did not specify which type was used [41–44]. Additionally, two studies utilized iRoot BP Plus [45] and iRoot SP (Innovative Bioceramix, Vancouver, Canada) [46]. These materials differ in both composition and radiopacifying agents, which can influence key factors such as biocompatibility, bioactivity, potential for discoloration, and overall clinical outcomes [40, 47].

Nevertheless, the literature on the success rates of clinical cases with extruded CS-based materials remains limited, with a predominant focus on initial endodontic therapy, especially with extrusion of MTA (Table 1) [41–43, 46, 48, 51–53]. In addition, the analysis of healing outcomes in the studies by Li et al. and Al Bakhakh et al. showed that the overall success rate between cases with extruded and nonextruded BC sealer (BC iRoot SP, TotalFill) did not demonstrate statistical significance [54, 55].

The healing results and absence of symptoms associated with the respective follow-up periods of this literature review are shown in Table 1.

Upon reviewing the data presented in Table 1, as these case reports were mostly traumatic dental injuries of teeth without apical constriction, certain factors must be considered that may have influenced the success and speed of periapical healing. Most cases were coated with CH prior to final obturation. Only Nagmode et al. and our last two case reports reported procedures with only one visit [42]. In addition, activated irrigation (e.g., PUI) was performed in a smaller number of cases. Since the average age of the patients in the analyzed case reports was 23.7 years, it is possible that patient age also plays a role in the healing of periapical lesions with extruded CS, although Li et al. found that age did not have a significant effect on the success of periapical healing [46].

Interestingly, in the research of Felippe et al., CH is listed as facilitating factor for accidental extrusion of MTA into periapical tissues due to formation of barriers beyond the apical foramen causing the difficulties in the formation of the apical plug [54]. In addition, factors such as overinstrumentation and excessive vertical pressure during the endodontic procedure may also contribute to RC sealer extrusion and should therefore be avoided [55].

It has been observed that in some cases (our case reports, Asgary et al., Nosrat et al.) reorganization and complete or partial resorption of the extruded CS material occurred along with periapical healing [49, 50]. These observations take time; therefore, when evaluating the success rate of periapical healing in such cases, it is recommended to extend the follow-up periods up to 7 years, as reported by some authors [50]. Therefore, the patients from our case reports will continue to be followed. This fact could be partially confirmed by the study of Nagmode et al. who observed only partial healing of the lesion after 6 months. It is possible that it would have been completed if the follow-up period had been longer [42].

Taking the abovementioned into account, most authors reported periapical healing despite extrusion, with the exception of three clinical cases that ended with periapical surgery [21, 44, 50]. Based on the data summarized in Table 1, it can be concluded that over 80% of the retreated cases showed complete healing of the periapical lesions, while the success rate in initially treated teeth was over 90%.

## 7. Conclusion

In conclusion, although various factors affect the healing process of the osseous tissue, this article emphasizes that extrusion of CS materials does not cause irritation of the periapical tissue in the majority of clinical cases but may even promote healing.

However, when evaluating the success rate of periapical healing in such cases, it is recommended to extend the follow-up periods to several years.

An appropriate application technique when manipulating with CS is desirable to optimize predictable outcomes and treatment success. Further studies with longer follow-up periods are required for extrusion of other BC materials, as a literature review revealed a predominance of case reports of extrusion with MTA.

## Figures and Tables

**Table 1:** Overview of the individual case reports with extruded CS materials and their healing outcome.

**Authors**	**No. of clinical cases**	**Type of CS sealer**	**Initial treatment (IT)/retreatment (RETX) (** **n** **)**	**Follow-up period**	**Periapical healing and absence of symptoms (** **n** **)**
Tezel et al., 2010 [48]	1	White ProRoot MTA^a^	IT	24 months	+
Tahan et al., 2010 [43]	1	ProRoot MTA (color nonspecified)^a^	IT	12 months	+
Asgary and Ehsani, 2012 [49]	1	Tooth-colored ProRoot MTA^a^	RETX	7 years	+
Brito-Junior et al., 2012 [44]	1	ProRoot MTA^a^ (color nonspecified)	RETX	6 months	−⁣^∗∗^
Nosrat et al., 2012 [49]	1	Tooth-colored ProRoot MTA^a^	IT	4 years	+
	1		RETX	27 months	−⁣^∗∗^
	1		RETX	12 months	−⁣^∗∗^
Chang et al., 2013 [41]	1	ProRoot MTA^a^ (color nonspecified)	IT	36 months	+
	1		RETX	54 months	+
	1		RETX	48 months	+
Nagmode et al., 2016 [42]	1	ProRoot MTA^a^ (color nonspecified)	RETX	6 months	+/−⁣^∗^
	2		IT	6 months	+/−⁣^∗^
Li et al., 2022 [46]	72	iRoot SP^b^	IT (55)	30.46 months	+ (52)
			RETX (17)		+ (17)
Nik-Aziz et al., 2022 [45]	1	iRoot BP Plus putty^b^	IT	12 months	+

^a^BC sealer manufacturer: ProRoot MTA, Dentsply Tulsa Dental, Tennessee, United States.

^b^BC sealer manufacturer: iRoot SP/iRoot BP Plus putty, Innovative Bioceramix, Vancouver, Canada.

⁣^∗^Gradual healing.

⁣^∗∗^Periradicular surgery.

## Data Availability

The authors confirm that the data supporting the findings of this study are available within the same article (“Periapically Extruded Calcium Silicate Cements in Retreated Teeth: A Case Report and Literature Review”).
